# Impact of scaling up harm reduction interventions on injecting risk behaviours, ART outcomes and HIV incidence among people who inject drugs in Kenya

**DOI:** 10.1016/j.drugpo.2025.104824

**Published:** 2025-05-05

**Authors:** Josephine G. Walker, Matthew J. Akiyama, Adelina Artenie, Charles M. Cleland, John A. Lizcano, Helgar Musyoki, Mercy Nyakowa, Peter Cherutich, Ann E. Kurth, Peter Vickerman

**Affiliations:** a University of Bristol, Bristol, United Kingdom; b Albert Einstein College of Medicine, Department of Medicine, New York, NY, United States; c New York University School of Medicine, Department of Population Health, New York, NY, United States; d New York Academy of Medicine, New York, NY, United States; e Yale University, Orange, CT, United States; f Kenya Ministry of Health, National AIDS & STI Control Program (NASCOP), Nairobi, Kenya

**Keywords:** People who inject drugs (PWID), Needle and syringe programs (NSPs), Antiretroviral therapy (ART), Opioid agonist therapy, Sub-Saharan Africa, Kenya, HIV

## Abstract

**Introduction:**

Little data exists on the effectiveness of HIV prevention interventions among people who inject drugs (PWID) in Africa. We used empirical data from Kenya to fill this evidence gap.

**Methods:**

Six rounds of bio-behavioural surveys using respondent-driven-sampling were conducted among PWID in Nairobi and Coastal Kenya over 2012–2015. Dried blood spot samples were tested for HIV and HIV viral load, and HIV incidence was estimated through linking participants between rounds. Regression analyses evaluated whether self-reported usage of opioid agonist therapy (OAT) or needle and syringe programmes (NSP) in last year were associated with reduced injecting risk behaviours, increased ART uptake and viral suppression, and reduced risk of HIV acquisition.

**Results:**

Overall, 4897 PWID participated in the study, with 3903 participating in >1 round. Over the rounds, coverage increased from zero to 80–86 % for NSP and zero to 10–20 % for OAT. The proportion of people living with HIV (PLHIV) that were virally suppressed increased from 7–14 % to 39–55 %. Accessing NSP and OAT was associated with reduced syringe sharing at last injection (NSP adjusted odds ratio (aOR)=0.31; 95 % CI:0.24–0.40; OAT aOR=0.046; 95 %CI:0.034–0.061) and OAT was associated with reduced injecting frequency (adjusted rate ratio=0.21; 95 %CI:0.12–0.36). Using OAT was associated with increased ART coverage (aOR=2.76; 95 %CI:1.50–5.06) and viral suppression (aOR=2.99; 95 %CI:1.78–5.03) among PLHIV, while NSP was not. HIV incidence decreased from 6.10 (95 %CI:3.56–9.77) to 1.49 (95 %CI:0.79–2.54) per 100 person-years between the first and second half of the study. Accessing NSP was associated with lower HIV incidence (adjusted hazard ratio=0.25; 95 %CI:0.087–0.58).

**Conclusions:**

This study provides strong evidence for the benefits of NSP and OAT on varied HIV outcomes among PWID in Africa.

## Introduction

Since 1999, the use of illegal drugs has expanded in Sub-Saharan Africa (Needle et al., 2006; Degenhardt et al., 2023), including Kenya (Beckerleg et al., 2005), with data suggesting there were 36,000 people who inject drugs (PWID) in Kenya in 2020, (Degenhardt et al., 2023) but possibly less in 2024 (14,000) (Kenya Ministry of Health, 2024). PWID are highly vulnerable for HIV infection, with an increasing number of HIV epidemics documented among PWID in sub-Saharan Africa (Degenhardt et al., 2023). The HIV prevalence among PWID in Kenya was estimated at 11.3 % in 2017–2019 (Degenhardt et al., 2023) and 9.1 % in 2024 (Kenya Ministry of Health, 2024); two times higher than the general population (4.8 % in 2018 (NACC, 2018)). Risk is further elevated in women who inject drugs, with data from Kenya and other African countries suggesting that they have much higher HIV prevalence than men who inject drugs (Kurth et al., 2015; Mmbaga et al., 2017; Lepretre et al., 2015).

Evidence from high income countries has shown that needle and syringe programmes (NSP) and opioid agonist therapy (OAT) can reduce the risk of HIV infection (Aspinall et al., 2014; MacArthur et al., 2012), while OAT can also improve outcomes for antiretroviral therapy (ART) (Low et al., 2016). However, despite PWID being a key population for HIV transmission, access to NSP and OAT is limited in SSA (Colledge-Frisby et al., 2023; Mbogo et al., 2022; Leyna et al., 2019).

The low coverage of services for PWID will partly be due to the criminalization of drug possession and use in most countries of Sub-Saharan Africa, making PWID a highly stigmatized group who are not considered a high priority by governments. Additionally, there is sparse empirical evidence on the benefits of OAT and NSP in Sub-Saharan Africa (Mbogo et al., 2022; Ubuguyu et al., 2016; Tran et al., 2015), likely hindering its expansion.

**Fig. 1.**
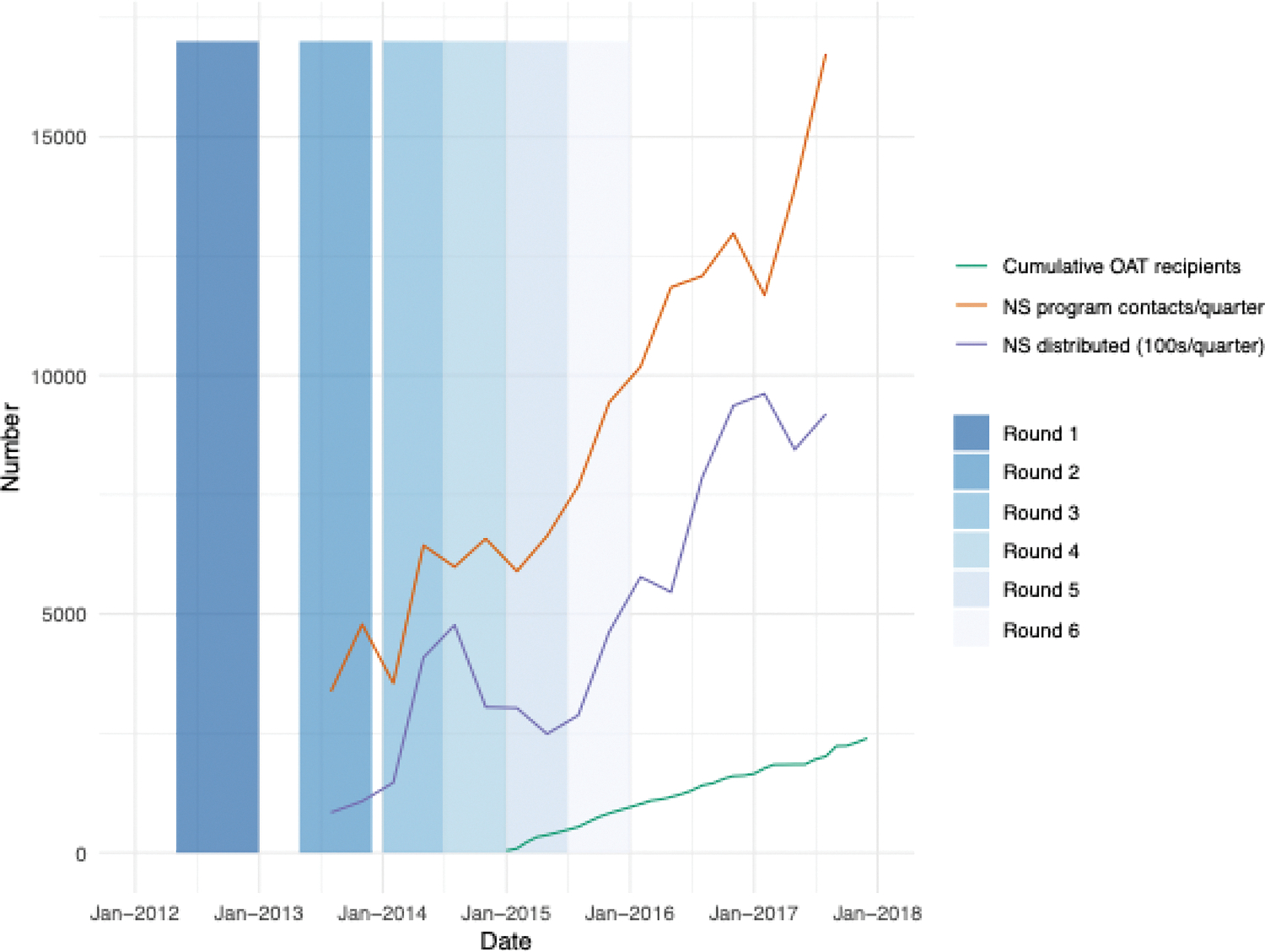
Changes over time in harm reduction access and corresponding timing of TLC-IDU study rounds. Vertical bars represent duration of each TLC-IDU bio-behavioural survey round. Lines show scale up of opioid agonist therapy (OAT) and needle and syringe (NS) distributions over time, based on programmatic data from NASCOP, NS numbers are presented quarterly, shown as midpoint of quarter, with NS distributed shown in 100 s. OAT numbers are cumulative - individuals ever enrolled on OAT.

The National AIDS & STI Control Program (NASCOP) initiated one of the first NSPs in Sub-Saharan Africa in November 2012 in Mombasa and April 2013 in Nairobi (Rhodes et al., 2015). These NSP services were scaled-up by community-based organizations (Fig. 1 (Stone et al., 2022)). An OAT program was initiated in Nairobi in December 2014, expanding to Coastal Kenya in 2015 (Rhodes et al., 2015), and scaling up from then (Fig. 1). In 2017, 189 needles were distributed per NSP user per year (NACC, 2018), and 3788 PWID were accessing OAT in 2021 (Stone et al., 2022). Although there is uncertainty around the uptake of ART and levels of viral suppression among PWID globally (Hajarizadeh et al., 2023), recent data from a national bio-behavioural survey in 2024 estimated 91 % of PWID on ART in Kenya were virally suppressed (200 copies/ml cut off (Kenya Ministry of Health, 2024)).

The Testing and Linkage to Care for Injecting Drug Users (TLC-IDU; PIs Cherutich and Kurth) study was initiated in May 2012 to improve linkage-to-care for PWID initiating ART in Kenya (Kurth et al., 2015). As part of this study, six rounds of bio-behavioural surveys were undertaken among PWID in Nairobi and Coastal Kenya over 2012–2015 corresponding to the time when NSP and OAT scaled up. To produce better evidence on the benefits of OAT and NSP in SSA to motivate their scale-up in SSA, we use data from the TLC-IDU studies to: 1) quantify the scale-up in NSP, OAT and ART over the study period; 2) determine whether NSP and OAT are associated with reductions in injecting risk behaviours and improved ART outcomes; and 3) evaluate whether these interventions was associated with reductions in HIV incidence.

## Methods

### Study sites and recruitment

Six rounds of cross-sectional bio-behavioural surveys were undertaken among PWID in Nairobi and Coastal Kenya over May 2012 to July 2015 (Fig. 1). Respondent-driven sampling (RDS) was used to recruit study participants in each survey. In each round, three to six initial seeds were recruited from 10 PWID-specific HIV prevention service sites to start the recruitment process. These service sites provided NSP and community-based outreach. Seeds recruited up to three peers that became study participants and recruiters for the next wave of RDS. This process continued resulting in several “waves” of recruitment.

Inclusion criteria for the RDS studies included: (1) ≥18 years old; (2) living in Nairobi or Coastal Kenya (Coast Province including Kwale, Mombasa, Kilifi counties); (3) lifetime history of injecting drugs; (4) report using any illicit, non-prescribed drugs by any route of administration in past year; and (5) able and willing to provide informed consent. In addition, we excluded those under the influence of substances, or if the interviewer was confident that the participant was not a PWID based on their survey responses and/or visual observation of the skin.

For each survey, respondent questionnaire data were collected by staff on tablet computers. The questionnaire (survey instrument included in the appendix) collected information on demographics and a risk assessment, which included HIV testing history, injecting and sexual risk behaviors, drug use, current housing status (unstable housing defined as being mobile when asked ‘how do you live?’), incarceration history (ever detained), exposure to PWID services and prevention methods for HIV—including OAT and NSP use in last year, and HIV treatment uptake. Collected data were transferred to a secure encrypted server.

### Sample testing

After completing the respondent questionnaire for each RDS round, rapid HIV testing (on finger prick blood sample) were undertaken for all study participants using Alere Determine HIV-1/2 Ag/Ab rapid test (Alere Inc., Waltham, MA, USA). Those testing positive underwent confirmatory testing using Uni-Gold HIV-1/2 (Trinity Biotech PLC, Wicklow, Ireland), and if non-reactive the Uni-Gold test was repeated, and the second result taken as the result.

To assess HIV viral load, we collected dried blood spot (DBS) specimens from all individuals identified as HIV-positive in each survey round. The National HIV Reference Laboratory used the COBAS AmpliPrep/COBAS TaqMan HIV-1 Qualitative Test, version 2.0 (CAP/CTM v2.0) to obtain HIV-1 viral load results. For all PWID testing HIV-positive, peer case managers facilitated linkage to care at a study-affiliated HIV clinic.

Participants received payment for completing the survey (350 Kenya shillings; Ksh), for returning to receive confirmatory HIV test results (250 Ksh; if the initial rapid test was positive), and for each successful referral of a peer in the RDS survey (250 Ksh).

### Statistical analysis

Descriptive data on the demographics, injecting risk behaviours, self-reported use of NSP and OAT in last year, and HIV prevalence by round and region (Coast or Nairobi) are presented accounting for RDS sampling weights using the RDS-II estimator due to it being more conservative than RDS-I (overestimating, rather than underestimating the variance) (Gile & Handcock, 2010). Among HIV-positive participants, self-reported use of ART, and level of viral suppression were also calculated by round and region with RDS sampling weighting. All analyses were done in R version 4.4.0.

We assessed whether self-reported usage of NSP and OAT in last year were associated with lower injecting risk behaviours in the overall pooled sample. This was done using Generalized Estimating Equations (GEE) models (Højsgaard et al., 2006) to account for clustering due to repeat participants across rounds (see below). Risk behaviours included whether the syringe used for last injection was previously used by someone else (yes/no; analyzed using a logistic model) and number of times injected in last 30 days (count; Poisson model). A small proportion of people did not inject in the last 30 days, 6.4 % in Coast and 7.3 % in Nairobi. GEE models were conducted assuming a first-order autoregressive correlation structure (Højsgaard et al., 2006) using the geepack package for the model where the outcome was injecting frequency. Penalized GEE using Firth’s method was used to provide bias-corrected finite estimates and sandwich estimates of the standard error for the model where the outcome is syringe re-use to account for data separation (no reported syringe sharing among OAT users). This was implemented using the geefirthr package (Geroldinger et al., 2022). The 95 % *CIs* from the Firth GEE were compared to standard GEE regression results without OAT included and all associations were very similar (Supplementary Table 9).

Among HIV-positive individuals, we also evaluated if self-reported usage of NSP and OAT were associated with improved HIV treatment outcomes. This was done using logistic GEE regression to look at the associations between access to OAT and/or NSP and currently being on ART (self-reported) or being virally suppressed (log10 viral load<3). We excluded rounds 1–2 due to the change in eligibility for ART by CD4 count in 2014 (CD4<350/μL before 2014 and <500/μL during 2014–2016 (rounds 3–6)).

All regression analyses adjusted for region, gender, duration of injecting, and unstable housing because previous analyses have found that these factors can affect both access to interventions and injecting risk behaviours or HIV transmission risk (Austin et al., 2021; Artenie et al., 2023; Arum et al., 2021). Incarceration history was not included in the injecting risk behaviour analyses as this question was not asked in rounds 1–2.

### HIV incidence

Individuals who participated in multiple rounds of the survey were matched based on their reported previous participation, birthdate, gender, and reported mother’s name; biometric matching was not used. Where matched individuals appeared to have participated multiple times in a specific survey round, we conservatively excluded them from the incidence analysis. We estimated HIV incidence among individuals who were HIV-negative at their first visit and participated in ≥2 rounds over the study period. New positive cases were assumed to occur halfway between the last negative and first positive tests. Other methods of estimating new HIV infections, such as recency testing, were not used. Incidence rates and Wald 95 % CI were calculated. We used Cox proportional hazard models with Firth’s penalized likelihood (coxphf package (Heinze et al., 2023), because there were no incident cases among those using OAT) to estimate the hazard ratios for the associations between HIV acquisition risk and region, gender, unstable housing, and whether NSP or OAT use is reported. NSP, OAT and unstable housing status were lagged one visit with respect to the outcome. Incarceration history was excluded due to not being measured in all rounds. Using Firth’s penalized likelihood did not allow for cluster-robust standard error estimation, but all profile penalized likelihood 95 %*CIs* were compared to standard Cox regression results accounting for clustering and all associations were very similar (Supplementary Table 10). In addition, we evaluated differences in incidence over time by stratifying the incidence data into the first three rounds (to June 2014) and last three rounds of the study. This analysis would have been done by survey round but there was insufficient data to do so.

### Ethical approvals

This study was approved by Kenyatta National Hospital, University of Nairobi Ethics and Research Committee P171/05/2011 and Yale University Institutional Review Board HIC#1512,016,965.

### Role of the funding source

The sponsor of the study had no role in study design, data collection, data analysis, data interpretation, or writing of the report. The authors had full access to all the study data and had final responsibility for the decision to submit for publication.

## Results

In total, 8800 questionnaire data points (8791 with HIV test results) were collected across the six rounds of RDS surveys, 4932 (56.0 %) in Coast and 3868 (44.0 %) in Nairobi. This included data from 4897 unique PWID, of whom 3903 (79.1 %) participated in >1 survey round. More than 93 % of respondents reported injecting in the last month; 281/3868 in Nairobi and 283/4932 in Coast did not inject in the last month. A total of 1319 viral load measures were taken among 1542 PWID testing HIV-positive in the study. Table 1 summarizes the demographic and other key factors of the participants who enrolled in the study. Across the survey rounds, most factors did not change substantially. Most (88.5 %) PWID were male, and the median duration of injecting was 3 years. The median injecting frequency was 60 injections per month, most PWID have been previously incarcerated (80.1 %), and one-quarter (23.0 %) were currently unstably housed. HIV prevalence gradually decreased from 19.5 % to 15.2 % over the survey rounds, mean age of PWID increased from 31.4 to 33.1 years, and the proportion that re-used a needle at last injection decreased from 10.6 % to 3.0 %.

### Intervention coverage and viral suppression

The proportion of participants self-reporting use of NSP in the last year increased substantially over the survey rounds in Nairobi and Coast from no provision in round 1 to ~80 % in round 6 (Table 2). The coverage of OAT in the last year and proportion of PLHIV self-reporting currently being on ART increased to a lesser extent, with OAT starting from round 5 and ART already having moderate coverage in round 1, especially in Coast. The proportion of PLHIV that were virally suppressed also increased but with some fluctuations. Levels of viral suppression among PLHIV that self-report being on ART were low in round 1 (18–21 %) and increased to moderate levels (45–62 %) by round 6 (Supplementary Table 1). For comparison, levels of viral suppression were lower among PLHIV that did not report being on ART (4–11 % in round 1) but also increased over the rounds (31–33 % in round 6). The prevalence of HIV in the study peaked in round 2 in Nairobi and then declined, while in Coast the prevalence declined over rounds 1 to 5 and then increased in round 6 (Table 1). It is uncertain whether this increase is meaningful because the confidence intervals overlap with the round 5 prevalence estimate.

### Associations between NSP, OAT and injecting risk behaviours and ART outcomes

Among all participants across both regions, accessing just NSP in the last year was associated with lower odds (aOR=0.31; 95 %CI 0.24–0.40) of using a previously used syringe at last injection (Table 3). OAT alone (aOR=0.046; 95 % CI 0.034–0.061) or in combination with NSP (aOR=0.026; 95 %CI 0.020–0.033) were also associated with reduced use of a previously used syringe; no individuals using OAT reported syringe sharing. The 95 %CI for these estimates may be overly narrow due to the limitations of the penalized GEE method (Geroldinger et al., 2022).

Compared to not accessing OAT and NSP, accessing just OAT in the last year was associated with much lower frequency of injecting in past 30 days (aRR=0.21; 95 %CI 0.12–0.36), as was accessing both OAT and NSP (aRR=0.56; 95 %CI 0.46–0.67), but to a lesser extent (Table 3). Lastly, accessing NSP alone without OAT was associated with a slightly higher frequency of injecting in last 30 days (aRR=1.13; 95 %CI 1.10–1.15) compared to using neither NSP and OAT.

Among PLHIV, using both OAT and NSP was positively associated with ART access (aOR=3.01; 95 %CI 1.34–6.79) and being virally suppressed (aOR=2.61; 95 %CI 1.35–5.05) across both regions (Table 4). Using just OAT was also marginally associated with increased ART access and viral suppression, but not significantly, while using just NSP was not associated. Importantly, when we undertook an additional regression analysis where OAT and NSP were incorporated as separate variables, any recent use of OAT was positively associated with ART access (aOR=2.76; 95 %CI 1.50–5.06) and being virally suppressed (aOR=2.99; 95 %CI 1.78–5.03), while recent use of NSP was not associated (Supplementary Table 6 and 7).

For these regression models, the adjusted associations for other variables are given in Supplementary Tables 2–5. For syringe sharing, we found it was lower in males than females, lower amongst stably housed PWID compared to unstably housed PWID, and was higher in Nairobi than Coastal region. For injecting frequency, it was lower amongst stably housed PWID, lower in Nairobi than Coastal region, and increased with injecting duration. For ART coverage, it was higher if they had ever been incarcerated, it increased with injecting duration and was higher in stably housed PWID. Similarly, levels of viral suppression increased with injecting duration and was higher if they were stably housed in unadjusted analysis, and were lower in Nairobi than Coastal region in adjusted analysis.

### Incidence of HIV

When accounting for repeated measures of individuals across rounds, there were 979 individuals that tested HIV-negative at initial participation in a survey round and that were in at least one further survey round (demographic details by round are summarised in Supplementary Table 8). Overall, 30 incident cases of HIV were observed among these individuals in a total time at risk of 1153 person-years (average follow-up of 1.18 person-years per participant), for a total HIV incidence of 2.6 per 100 person-years (Wald 95 %CI 1.76–3.71). In Coast, the incidence was 1.84 per 100 person-years (15 cases/814 person-years), compared to 4.42 per 100 person-years in Nairobi (15 cases/338 person-years). Across both regions, incidence was higher in women than men with 6 incident cases in women over 102 person-years (5.86 per 100 person-years) and 24 incident cases in men over 1050 person-years (2.29 per 100 person-years). Kaplan-Meier curves for region and gender are shown in Supplementary Figure 1. In the first 3 rounds of the study, 17 incident cases occurred in 279 person-years for an incidence rate of 6.1 (95 %CI 3.56–9.77) per 100 person-years, while in the last 3 rounds of the study, there were 13 incident cases in 875 person-years (incidence rate 1.49, 95 %CI 0.79–2.54 per 100 person-years).

The hazard ratios (HR) are shown in Table 5. Over the full time-period, there was a lower hazard of HIV acquisition associated with male gender (aHR=0.29; 95 %CI 0.13–0.71) and accessing NSP in last year (aHR=0.25; 95 %CI 0.09–0.58). No incident HIV infections occurred among PWID accessing OAT, but we only had 2.87 person-years of follow-up time for OAT alone and 5.14 person-years for OAT and NSP together, compared to >500 person-years for NSP only or neither. We observed a higher hazard of HIV acquisition associated with being in Nairobi in unadjusted analysis only (HR=2.18, 95 %CI 1.07–4.45; aHR=2.20, 95 %CI 0.90–5.12) and tendency for reduced incidence in PWID with stable housing in unadjusted analysis only (HR=0.47, 95 %CI 0.23–1.02).

## Discussion

This study leveraged a national scale-up in harm reduction interventions among PWIDs in Kenya to ascertain their real-world impact on multiple outcomes. Our analysis produces evidence for the benefits of OAT and NSP in reducing injecting risk behaviours, improving ART uptake and viral suppression and for NSP reducing HIV incidence among PWID. Specifically, accessing NSP and/or OAT are shown to be associated with reduced syringe sharing (by over two-thirds) while using OAT is associated with reduced injecting frequency (by over two-fifths). Accessing OAT is also associated with greater ART uptake and viral suppression (about three-times), but NSP is not, while accessing NSP is associated with a large reduction (by three-quarters) in the risk of HIV acquisition. Importantly, HIV incidence also reduced by three-quarters over the study period, which occurred while OAT, NSP and ART scaled up among PWID in these settings. This decrease, together with our findings that PWID receiving OAT and NSP had lower HIV risk and improved HIV treatment outcomes, strongly suggest that the scale-up of these interventions have decreased the incidence of HIV among PWID in Kenya.

Other than these main findings, we also found other important associations. These include females having three times higher HIV incidence than men and higher rates of syringe sharing. Female PWID also reported high rates of sex work in our dataset (67 %, analysis not shown), suggesting that their heightened HIV incidence may be due to increased sexual and injecting risk. This heightened HIV risk among females is much higher than was found in our recent systematic review (Artenie et al., 2023), highlighting the added vulnerability that female PWID experience in SSA. In line with these findings, studies from SSA have also found much higher HIV prevalences and increased levels of sexual risk behaviours among female PWID than male PWID (Kurth et al., 2015; Stone et al., 2022; Bowring et al., 2012). This emphasizes the need for sex-specific HIV programming for PWID in SSA, with these interventions also needing to focus on the sexual risk behaviours of female PWID (Stone et al., 2022). Additionally, although the HIV prevalence was higher in the Coastal region, we found a two-fold higher HIV incidence in Nairobi, aligning with Nairobi having lower levels of viral suppression and increased syringe sharing. Although the reasons for these differences in HIV intervention outcomes are uncertain, the lower levels of viral suppression in Nairobi may partly be due to their later scale-up of ART compared to the Coastal region. Lastly, as found in other studies, we found that unstably housed PWID had higher injecting frequency, higher levels of syringe sharing, lower ART coverage and lower levels of viral suppression (Genberg et al., 2019; Topp et al., 2013; Corneil et al., 2006). HIV prevention services need to account for this pervasive structural factor because it is linked to so many deleterious health outcomes among PWID.

### Strengths and limitations

The strength of our analysis lies in its novelty, in producing much needed evidence from SSA on the benefits of OAT and NSP on multiple outcomes, and in producing one of the first estimates for the incidence of HIV among PWID in SSA.

Limitations include not undertaking a randomized controlled trial to assess the impact of OAT and NSP on different outcomes. Unfortunately, this type of study is not possible for ethical reasons because OAT and NSP have been shown to have many benefits in other regions. Instead, we utilized an observational study design, which means we cannot rule out the possibility that some of our associations are due to confounding. Although we tried to minimize this possibility by controlling for likely confounders, we cannot say with certainty that our associations imply a causal mechanism.

The RDS recruitment approach allows for recruitment of hard to reach populations, but is biased due to different probabilities of recruitment for individuals depending on their network size. This bias was adjusted for in our analyses. Many of our analyses were dependent on self-reported data on injecting risk behaviours and uptake of interventions. Although these data could be subject to social desirability bias, especially the data on syringe sharing, we feel the consistency of our findings, including those that utilise biological measures, improves the confidence in our findings. Also, other studies have shown that self-reported data are likely to be reliable (Darke, 1998). Using a self-reported measure of ART use is a limitation because some individuals will not divulge their HIV status. This limitation was counteracted by having tested viral suppression data, with this data suggesting that some PLHIV not reporting being on ART were on ART because a fair proportion were virally suppressed. Our housing question was also limited because the survey only included an option for someone being ‘mobile’ when asked ‘how do you live?’. Although, it is possible that this answer could refer to someone migrating between towns or having multiple homes, it most likely captures people having unstable living conditions. Limitations of Firth’s penalized regression method to correct for separable data within GEEs and Cox regression mean that our estimates of the association between OAT use and syringe sharing or HIV incidence should be interpreted with caution.

HIV incidence was estimated through linking HIV test results for people that participated in multiple survey rounds, which was a subset of all participants. These people were identified through using survey responses to match people across rounds. Although this linkage could have been done more effectively through using biometric data, as done by others (Clipman et al., 2024), care has to be taken collecting such data because it can be misused to identify individuals involved in drug use, which is criminalised in Kenya. People who participated in multiple rounds were found to differ from those that did not by having lower likelihood of syringe sharing (1.6 % versus 4.6 % in last injection), lower levels of unstable housing (17.6 % versus 23.0 %), and being less likely to be female (8.3 % versus 11.4 %) or from Nairobi (33.6 % versus 44.0 %; comparing Table 1 to Supplementary Table 8). Although these differences suggest that our estimated incidence of HIV is for a sub-group of PWID with lower HIV-risk, we do not think it affected our findings on how NSP uptake is associated with HIV incidence.

Lastly, the survey data used in our study is relatively old, dating from 2012 to 2015. We do not think this reduces the relevance of our analysis because it produces much needed evidence on the benefits of harm reduction interventions in SSA. Indeed, the roll-out of harm reduction interventions are still at an early stage in SSA (Colledge-Frisby et al., 2023) and so this evidence is still highly policy relevant for advocating for their expansion in other African countries.

### Comparison with other studies

Other studies have documented the impact of harm reduction interventions among PWID, but few studies are from LMICs and Africa. Previous systematic reviews synthesizing evidence on the impact of NSP and OAT on HIV incidence (Aspinall et al., 2014; MacArthur et al., 2012) or OAT on ART outcomes (Low et al., 2016) have largely depended on studies from high-income countries (4 of >50 studies in previous systematic reviews were from LMIC) with no studies from Africa. Since these reviews, some small-scale studies (<700 participants in each study) from Africa (Tanzania, Kenya and Egypt) have produced evidence for the benefits of OAT or NSP on different HIV or HCV-related outcomes (Mmbaga et al., 2017; Mbogo et al., 2022; Leyna et al., 2019; Ghaddar & Ghaly, 2016; Hassan et al., 2019; Likindikoki et al., 2020). This includes evidence that being on OAT and/or having access to clean syringes (proxy for NSP) improved ART uptake and viral suppression (just OAT (Mbogo et al., 2022; Hassan et al., 2019)), reduced syringe sharing (just NSP (Ghaddar & Ghaly, 2016)), and decreased odds of prevalent HIV or HCV infection (just NSP (Mmbaga et al., 2017; Leyna et al., 2019; Likindikoki et al., 2020)). However, no studies from Africa have considered the effect of OAT and NSP on HIV or HCV incidence. Our large-scale study builds on these previous studies by providing robust and consistent evidence for the benefits of OAT and NSP on HIV incidence and a range of other outcomes. In terms of presenting data on HIV incidence among PWID, only one study by our group has provided estimates of HIV incidence among PWID for sub-Saharan Africa, for South Africa (Artenie et al., 2024), while very few studies globally have documented how HIV incidence can decrease among PWID following the scale-up of interventions (Huang et al., 2014). Our study provides valuable data in this area, illustrating the high HIV incidence that can exist among PWID before the scale-up in interventions, and showing how that can decrease substantially when they are scaled up.

### Implications

Our study shows the impact that harm reduction interventions can have in Africa. Kenya is already seen as an important case study on how interventions should be rolled out for PWID in Africa (UNAIDS, 2019). Our study adds evidence to this by showing the success of what they have achieved. This evidence is important for Kenya to advocate for further scale-up of OAT, where coverage is still sub-optimal (Colledge-Frisby et al., 2023; Stone et al., 2022), as well as for maintaining the current high coverage of NSP. For other countries in Africa, our findings show the impact that harm reduction interventions can have, providing valuable evidence to persuade policy makers to initiate and roll out these services in their countries. Low levels of viral suppression among PWID who self-report being on ART also highlight that extra efforts may be needed among PWID to ensure sufficient adherence. Encouragingly, recent data from Kenya shows that PWID on ART now have high levels of viral suppression (91 %) (Kenya Ministry of Health, 2024). Lastly, our findings of high HIV incidence and syringe sharing among female PWID, as well as poor outcomes among unstably housed PWID emphasize the need for specific strategies to focus on these and other vulnerable groups in Kenya and Africa (Arum et al., 2021; Ayon et al., 2019). Without doing this, women who inject drugs and unstably housed PWID are likely to remain at heightened risk even with the successful scale-up of harm reduction interventions for PWID.

## Supplementary Material

Supplementary Materials

Supplementary materials

Supplementary material associated with this article can be found, in the online version, at doi:10.1016/j.drugpo.2025.104824.

## Figures and Tables

**Table 1:** Demographic and key characteristics of PWID that participated in each survey round. Overall, **4897** unique PWID participated in one or more survey rounds, of which **3903** participated in more than one, with there being **8800** total interviews across the surveys. The table summarises those who participated. Round 1: May 2012-Dec 2012. Round 2: April 2013-Nov 2013; Round 3: Jan 2014-Jun 2014; Round 4: July 2014-Dec 2014; Round 5: Jan 2015-Jun 2015; Round 6: July 2015-Dec 2015.

Survey round	1	2	3	4	5	6	Total

**Screened**	1946	1739	1265	1336	1460	1677	9423
**Number interviewed in survey**	1785	1489	1186	1286	1395	1659	8800
**First time participating**	1785, 100 %	1434, 96.3 %	710, 59.9 %	705, 54.8 %	722, 51.8 %	968, 58.3 %	
Region							
Coast	1122 (62.9 %)	873 (58.6 %)	671 (56.6 %)	673 (52.3 %)	751 (53.8 %)	842 (50.8 %)	4932 (56.0 %)
Nairobi	663 (37.1 %)	616 (41.4 %)	515 (43.4 %)	613 (47.7 %)	644 (46.2 %)	817 (49.2 %)	3868 (44.0 %)
Gender							
Female	236 (13.2 %)	180 (12.1 %)	108 (9.1 %)	150 (11.7 %)	148 (10.6 %)	189 (11.4 %)	1011 (11.5 %)
Male	1549 (86.8 %)	1309 (87.9 %)	1078 (90.9 %)	1136 (88.3 %)	1247 (89.4 %)	1470 (88.6 %)	7789 (88.5 %)
Age							
Mean (SD)	31.4 (6.8)	31.6 (7.1)	32.2 (6.7)	32.1 (6.9)	33.2 (6.9)	33.1 (7.1)	32.3 (7.0)
Min - Max	18– 77	18– 83	18– 69	18– 61	18– 66	18– 67	18– 83
Unstable housing							
I am mobile	359 (20.1 %)	377 (25.3 %)	295 (24.9 %)	392 (30.5 %)	344 (24.7 %)	257 (15.5 %)	2024 (23.0 %)
I stay in one place	1426 (79.9 %)	1112 (74.6 %)	891 (75.1 %)	894 (69.5 %)	1051 (75.3 %)	1402 (84.5 %)	6776 (77.0 %)
Ever imprisoned*							
No	–	–	126 (18.5 %)	237 (18.4 %)	240 (17.2 %)	397 (23.9 %)	1000 (19.9 %)
Yes	–	–	555 (81.5 %)	1049 (81.6 %)	1155 (82.8 %)	1262 (76.1 %)	4021 (80.1 %)
Missing	1785	1489	505	0	0	0	3779
Years injecting							
Median (Q1, Q3)	3 (2,8)	2.5 (1, 6)	3 (1, 7)	3 (1, 6)	4 (2, 7)	3 (2, 6)	3 (1, 6)
Missing	0	7	2	2	7	4	22
HIV-status							
Negative	1435 (80.4 %)	1198 (80.5 %)	956 (80.8 %)	1074 (83.5 %)	1179 (84.5 %)	1407 (84.8 %)	7249 (82.4 %)
Positive	348 (19.5 %)	290 (19.5 %)	225 (19.0 %)	212 (16.5 %)	215 (15.4 %)	252 (15.2 %)	1542 (17.5 %)
Indeterminate	2 (0.1 %)	1 (0.1 %)	2 (0.2 %)	0 (0.0 %)	1 (0.1 %)	0 (0.0 %)	6 (0.1 %)
Missing	0	0	3	0	0	0	3
Times injected in last 30 days							
Mean (SD)	68.6 (33.1)	71.2 (34.1)	70.8 (37.2)	75.6 (33.0)	71.0 (38.6)	68.3 (40.6)	70.7 (36.28)
Median (Q1-Q3)	60 (60 –90)	60 (60 – 90)	63 (60–90)	90 (60 –90)	80 (60– 90)	60 (55–90)	60 (60–90)
Used previously used needle at last injection							
No	1590 (89.1 %)	1429 (96.0 %)	1158 (97.6 %)	1251 (97.3 %)	1348 (96.6 %)	1609 (97.0 %)	8385 (95.3 %)
Yes	190 (10.6 %)	59 (4.0 %)	27 (2.3 %)	34 (2.6 %)	46 (3.3 %)	50 (3.0 %)	406 (4.6 %)
Don’t know	5 (0.3 %)	1 (0.1 %)	1 (0.1 %)	1 (0.1 %)	1 (0.1 %)	0 (0.0 %)	9 (0.1 %)

* Ever imprisoned was not asked in rounds 1 and 2.

**Table 2:** Coverage of opioid agonist therapy (OAT), needle and syringe programmes (NSP), ART (all self-reported), HIV prevalence and levels of viral suppression (tested amongst those testing HIV positive) for Nairobi and Coastal region. Percentages for NSP, OAT, HIV prevalence, ART coverage and levels of viral suppression are RDS weighted with 95 % confidence intervals.

	NSP in last 12 months	OAT in last 12 months	HIV positive	Currently on ART^^^	Currently virally suppressed*^,^^

Nairobi					
Round 1 (*n* = 663)	0	0	113 (14.4 %, 11.2–17.6 %)	9 (16.5 %, 8.8–24.2 %)	6 (13.8 %, 0.9–26.7 %)
Round 2 (*n* = 616)	39 (5.5 %, 2.6–8.4 %)	0	125 (18.8 %, 14.2–23.4 %)	20 (30.6 %, 15.6–45.5 %)	15 (11.0 %, 4.1–17.9 %)
Round 3 (*n* = 515)	358 (68.7 %, 64.6–72.8 %)	0	101 (18.4 %, 14.7–22.1 %)	29 (33.2 %, 23.4–42.9 %)	23 (21.2 % (13.9–28.5 %)
Round 4 (*n* = 613)	406 (64.2 %, 61.7–75.7 %)	1 (0.15 %, 0.13–0.19 %)	98 (16.7 %, 14.5–18.8 %)	31 (41.1 %, 34.1–48.0 %)	18 (23.7 %, 15.3–32.0 %)
Round 5 (*n* = 644)	468 (73.2 %, 70.8–75.7 %)	102 (13.9 %, 12.3–15.5 %)	105 (14.9 %, 13.0–16.8 %)	51 (47.9 %, 42.6–53.2 %)	28 (25.3 %, 21.0–29.6 %)
Round 6 (*n* = 817)	661 (79.7 %, 76.7–82.8 %)	167 (19.9 %, 15.5–24.4 %)	121 (12.3 %, 10.4–14.2 %)	73 (65.7 %, 60.3–71.0 %)	47 (37.8 %, 30.8–44.8 %)
Coastal Region					
Round 1 (*n* = 1122)	0	0	235 (20.4 %, 17.5–23.3 %	91 (51.8 %, 43.0–60.7 %)	33 (15.4 %, 8.4–22.3 %)
Round 2 (*n* = 873)	59 (6.7 %, 4.5–8.9 %)	0	165 (17.9 %, 14.5–21.3 %)	58 (50.9 %, 38.9–63.0 %)	22 (14.8 %, 6.9–22.7 %)
Round 3 (*n* = 671)	412 (54.4 %, 49.3–59.6 %)	0	124 (17.0 %, 14.3–19.8 %)	60 (50.4 %, 40.7–60.1 %)	51 (40.6 %, 31.4–49.9 %)
Round 4 (*n* = 673)	543 (78.3 %, 74.8–81.8 %)	0	114 (16.9 %, 13.4–20.5 %)	52 (54.4 %, 41.0–67.8 %)	22 (29.3 %, 13.2–45.4 %)
Round 5 (*n* = 751)	609 (82.5 %, 80.1–84.8 %)	5 (0.41 %, 0.36–0.47 %)	110 (15.2 %, 11.3–19.0 %)	67 (64.0 %, 49.9–78.1 %)	36 (38.8 %, 23.8–53.8 %)
Round 6 (*n* = 842)	716 (85.5 %, 82.1–88.0 %)	73 (9.6 %, 7.5–11.8 %)	131 (18.3 %, 14.1–22.4 %)	89 (64.3 %, 50.1–78.4 %)	72 (49.0 %, 36.1–61.9 %)

^ Of those HIV positive although denominator is not same as number HIV-positive;.

* log10 VL < 3.

**Table 3:** Associations between opioid agonist therapy (OAT) and needle and syringe programme (NSP) access in the last 12 months, and injecting risk behaviours. Odds ratios (OR) calculated for binary outcomes using Firth’s penalization for bias correction in Generalized Estimating Equation (GEE). Rate ratios (RR) for GEE Poisson regression result. Adjusted models include region, gender, unstable housing, and duration of injection. Full results for each model and standard GEE results for the logistic regression are in supplementary Tables 2 and 3.

Outcome	Exposure	Unadjusted odds ratio (OR; 95 % CI)	Adjusted aOR (95 % CI)

Used previously used syringe at last injection	Neither OAT nor NSP (reference)	1	1
	NSP only	0.30 (0.23–0.39)	0.31 (0.24–0.40)
	OAT only	0.072 (0.056–0.092)	0.046 (0.034–0.061)
	Both NSP and OAT	0.026 (0.022–0.032)	0.026 (0.020–0.033)
		**Unadjusted rate ratio (RR; 95 % CI)**	**Adjusted aRR (95 % CI)**
Number of times injected in last 30 days	Neither OAT nor NSP (reference)	1	1
	NSP only	1.11 (1.09–1.14)	1.13 (1.10–1.15)
	OAT only	0.21 (0.12–0.36)	0.21 (0.12–0.36)
	Both NSP and OAT	0.53 (0.44–0.64)	0.56 (0.46–0.67)

**Table 4:** Associations between opioid agonist therapy (OAT) and needle and syringe programme (NSP) access and being on ART or viral suppression among people living with HIV from both regions (only rounds 3–6 due to change in ART eligibility requirements); odds ratios (OR) from Generalized Estimating Equations (GEE) logistic regression. Models adjusted for region, gender, duration of injection, unstable housing, and incarceration history. Full results (OR) for each model are in supplementary Table 4 and 5.

Outcome	Exposure	Unadjusted odds ratio (OR; 95 % CI)	Adjusted aOR (95 % CI)

Viral suppression (log10 VL < 3 versus ≥3)	Neither OAT nor NSP (reference)	1	1
	NSP only	0.81 (0.56–1.17)	0.75 (0.51–1.10)
	OAT only	1.70 (0.64–4.52)	2.00 (0.75–5.37)
	Both	2.59 (1.38–4.89)	2.61 (1.35–5.05)
Currently on ART	Neither OAT nor NSP (reference)	1	1
	NSP only	0.93 (0.65–1.34)	0.89 (0.60–1.30)
	OAT only	1.44 (0.51–4.08)	1.62 (0.61–4.30)
	Both	3.61 (1.70–7.66)	3.01 (1.34–6.79)

**Table 5:** Hazard ratios (HR) for associations with HIV acquisition risk by Cox regression with Firth’s penalized maximum likelihood, standard Cox model results are in supplementary Table 10.

Variable	Unadjusted HR (95 % CI)	Adjusted aHR (95 % CI) (full model)

Male gender (reference: female)	0.28 (0.13–0.69)	0.29 (0.13–0.71)
Nairobi region (reference: Coast)	2.18 (1.07–4.45)	2.20 (0.90–5.12)
NSP only (reference: neither)	0.26 (0.093–0.63)	0.25 (0.087–0.58)
OAT only (reference: neither)	3.61 (0.028–25.94)	1.69 (0.013–13.08)
Both NSP and OAT (reference: neither)	2.27 (0.018–16.75)	1.42 (0.011–12.07)
Stable housing (reference: unstable housing)	0.47 (0.23–1.02)	0.77 (0.31–1.95)

OAT: opioid agonist therapy; NSP: needle and syringe programme.
