# Supplementary material for: Impact of scaling up harm reduction interventions on injecting risk behaviours, ART outcomes and HIV incidence among people who inject drugs in Kenya
Source: Int J Drug Policy. Author manuscript; Available in PMC 2026 Jan 31. (PMC12860403; doi:10.1016/j.drugpo.2025.104824)
Supplement: Supplementary Materials [file NIHMS2132265-supplement-Supplementary_Materials.docx]

**Supplementary Tables and Figures**

**Supplementary Table 1**: Levels of viral suppression for PLHIV that self-report being on ART and those that do not report being on ART for Nairobi and Coastal region. Percentages for levels of viral suppression are RDS weighted with 95% confidence intervals (CI) except for ** entry. RDS-II weighting is used, however the analytical variance calculation can lead to CI below 0 when sample size is small and percentage is low, where this is the case the lower bound of the CI has been set to 0 and indicated by *.

| Round | Nairobi – on ART | Nairobi – not ART | Coast – on ART | Coast – not ART |
| --- | --- | --- | --- | --- |
| 1 | 1/5  (18.2%, 0*-48.6%) | 2/38  (4.3%, 2.9-5.8%) | 17/84  (21.0%, 8.0-34.0%) | 9/74  (10.7%, 3.1-18.3%) |
| 2 | 3/20  (12.1%, 0*-24.9%) | 10/69  (13.4% 2.6-24.2%) | 11/57  (21.4%,4.6-38.2%) | 7/72  (8.6%, 5.8-11.4%) |
| 3 | 13/29  (39.8%, 22.8-56.8%) | 9/53  (16.7%, 6.7-26.4%) | 33/59  (53.6%, 40.8-66.3%) | 14/47  (28.5%, 16.4-40.7%) |
| 4 | 10/26  (40.7%, 31.4-50.1%) | 5/40  (12.5%, 8.4-16.5%) | 17/44  (41.2%, 24.3-58.1%) | 4/45  (16.7%, 0*-51.0%) |
| 5 | 16/48  (33.3%, 20.4-8.4%)** | 9/41  (16.7%, 9.0-24.4%) | 28/61  (53.3%, 33.6-73.1%) | 7/30  (22.7%, 7.8-37.6%) |
| 6 | 34/70  (45.4%, 36.2-54.6%) | 13/41  (30.6%, 22.4-38.3%) | 57/85  (62.0%, 47.6-76.3%) | 13/31  (33.4%, 11.3-55.5%) |

****** Not RDS weighted - RDS weighted estimate could not be produced because the estimated network population size is lower than the subset being looked at

**Supplementary Table 2**: Generalized Estimating Equations (GEE) logistic regression results for variables associated with outcome “used previously used syringe at last injection”. The odds ratio (OR) for NSP or OAT access and multi-variable model were conducted using Firth’s penalization for bias correction to account for separation (due to no reported use of previously used syringes among those using OAT). Bias-corrected standard errors were used to calculate 95% CI. Analysis conducted on complete cases (6,190 clusters with maximum size 6).

| **Variable** | **Unadjusted OR (95% CI)** | **Adjusted aOR (95% CI)** |
| --- | --- | --- |
| Neither OAT or NSP access (reference) | 1 | 1 |
| NSP only in last 12 months | 0.30 (0.23-0.39) | 0.31 (0.24-0.40) |
| OAT only in last 12 months | 0.072 (0.056-0.092) | 0.046 (0.034-0.061) |
| Both OAT and NSP in last 12 months | 0.026 (0.022-0.032) | 0.026 (0.020-0.033) |
| Nairobi region (reference is Coastal region) | 2.44 (1.95-3.05) | 1.93 (1.52-2.45) |
| Male gender (reference is female) | 0.63 (0.47-0.83) | 0.70 (0.53-0.93) |
| Stably housed (reference is unstable housing) | 0.36 (0.29-0.44) | 0.56 (0.45-0.70) |
| Injecting duration in years | 1.00 (0.99-1.02) | 1.02 (1.00-1.03) |

NSP: needle and syringe programme

**Supplementary Table 3**: Generalized Estimating Equations (GEE) Poisson regression results for variables associated with outcome “number of times injected in last 30 days” Analysis conducted on complete cases (6,190 clusters with maximum size 6).

| **Variable** | **Unadjusted RR (95% CI)** | **Adjusted aRR (95% CI)** |
| --- | --- | --- |
| Neither OAT or NSP access (reference) | 1 | 1 |
| NSP only in last 12 months | 1.11 (1.09-1.14) | 1.13 (1.10-1.15) |
| OAT only in last 12 months | 0.21 (0.12-0.36) | 0.21 (0.12-0.36) |
| Both OAT and NSP in last 12 months | 0.53 (0.44-0.64) | 0.56 (0.46-0.67) |
| Male gender (reference is female) | 1.02 (0.97-1.06) | 1.00 (0.96-1.04) |
| Nairobi region (reference is Coastal region) | 0.95 (0.92-0.97) | 0.92 (0.90-0.95) |
| Duration of injecting (years) | 1.00 (1.00-1.01) | 1.01 (1.00-1.01) |
| Stably housed (reference is unstable housing) | 0.90 (0.88-0.93) | 0.87 (0.85-0.90) |

OAT: opioid agonist therapy; NSP: needle and syringe programme

**Supplementary Table 4:** Generalized Estimating Equations (GEE) logistic regression results for variables associated with self-reporting being on ART among people living with HIV. Analysis conducted on complete cases.

| **Variable** | **Unadjusted aOR (95% CI)** | **Adjusted aOR (95% CI)** |
| --- | --- | --- |
| Neither OAT or NSP (reference) | 1 | 1 |
| NSP only in last 12 months | 0.93 (0.65-1.34) | 0.89 (0.60-1.30) |
| OAT only in last 12 months | 1.44 (0.51-4.08) | 1.62 (0.61-4.30) |
| Both OAT and NSP in last 12 months | 3.61 (1.70-7.66) | 3.01 (1.34-6.79) |
| Nairobi region (reference is Coastal region) | 0.76 (0.56-1.03) | 0.95 (0.66-1.39) |
| Duration of injecting (years) | 1.05 (1.02-1.08) | 1.04 (1.02-1.07) |
| Stably housed (reference is unstable housing) | 2.27 (1.59-3.24) | 2.04 (1.34-3.11) |
| Male gender (reference is female) | 1.38 (0.98-1.94) | 1.18 (0.83-1.69) |
| Ever incarcerated (reference is never incarcerated) | 1.81 (1.16-2.82) | 1.64 (1.05-2.56) |

OAT: opioid agonist therapy; NSP: needle and syringe programme

**Supplementary Table 5:** Generalized Estimating Equations (GEE) logistic regression results for variables associated with being virally suppressed among people living with HIV. Analysis conducted on complete cases (700 clusters with maximum size 2).

| **Variable** | **Unadjusted aOR (95% CI)** | **Adjusted aOR (95% CI)** |
| --- | --- | --- |
| Neither OAT or NSP (reference) | 1 | 1 |
| NSP only in last 12 months | 0.81 (0.56-1.17) | 0.75 (0.51-1.10) |
| OAT only in last 12 months | 1.70 (0.64-4.52) | 2.00 (0.75-5.37) |
| Both OAT and NSP in last 12 months | 2.59 (1.38-4.89) | 2.61 (1.35-5.05) |
| Nairobi region (reference is Coastal region) | 0.76 (0.37-1.55) | 0.68 (0.47-0.98) |
| Duration of injecting (years) | 1.03 (1.00-1.05) | 1.02 (1.00-1.05) |
| Stably housed (reference is unstable housing) | 1.83 (1.25-2.69) | 1.39 (0.89-2.17) |
| Male gender (reference is female) | 1.03 (0.30-3.50) | 0.93 (0.65-1.33) |
| Ever incarcerated (reference is never incarcerated) | 1.18 (0.47-2.98) | 1.04 (0.65-1.64) |

OAT: opioid agonist therapy; NSP: needle and syringe programme

**Supplementary Table 6:** Generalized Estimating Equations (GEE) logistic regression results for variables associated with self-reporting being on ART among people living with HIV. Analysis conducted on complete cases (700 clusters with maximum size 2).

| **Variable** | **Unadjusted aOR (95% CI)** | **Adjusted aOR (95% CI)** |
| --- | --- | --- |
| NSP in last 12 months (reference is no NSP) | 1.02 (0.72-1.44) | 0.94 (0.65-1.36) |
| OAT in last 12 months (reference is no OAT) | 2.95 (1.66-5.25) | 2.76 (1.50-5.06) |
| Nairobi region (reference is Coastal region) | 0.76 (0.56-1.03) | 0.95 (0.66-1.39) |
| Duration of injecting (years) | 1.05 (1.02-1.08) | 1.04 (1.01-1.07) |
| Stably housed (reference is unstable housing) | 2.27 (1.59-3.24) | 2.07 (1.35-3.16) |
| Male gender (reference is female) | 1.38 (0.98-1.94) | 1.20 (0.84-1.71) |
| Ever incarcerated (reference is never incarcerated) | 1.81 (1.16-2.82) | 1.67 (1.07-2.62) |

OAT: opioid agonist therapy; NSP: needle and syringe programme

**Supplementary Table 7:** Generalized Estimating Equations (GEE) logistic regression results for variables associated with being virally suppressed among people living with HIV. Analysis conducted on complete cases (700 clusters with maximum size 2).

| **Variable** | **Unadjusted aOR (95% CI)** | **Adjusted aOR (95% CI)** |
| --- | --- | --- |
| NSP in last 12 months (reference is no NSP) | 0.87 (0.61-1.23) | 0.79 (0.55-1.14) |
| OAT in last 12 months (reference is no OAT) | 2.67 (1.26-5.65) | 2.99 (1.78-5.03) |
| Nairobi region (reference is Coastal region) | 0.76 (0.37-1.55) | 0.68 (0.47-0.98) |
| Duration of injecting (years) | 1.03 (1.00-1.05) | 1.02 (1.00-1.05) |
| Stably housed (reference is unstable housing) | 1.83 (1.25-2.69) | 1.40 (0.90-2.19) |
| Male gender (reference is female) | 1.03 (0.30-3.50) | 0.94 (0.66-1.35) |
| Ever incarcerated (reference is never incarcerated) | 1.18 (0.47-2.98) | 1.05 (0.66-1.67) |

OAT: opioid agonist therapy; NSP: needle and syringe programme

**Supplementary Table 8: Description of incidence dataset**

| Survey round | 1 (N=68) | 2 (N=460) | 3 (N=506) | 4 (N=549) | 5 (N=617) | 6 (N=498) | Total (N=2698) | p-value |
| --- | --- | --- | --- | --- | --- | --- | --- | --- |
| **Region** |  |  |  |  |  |  |  |  |
| Coast | 46 (67.6%) | 329 (71.5%) | 329 (65.0%) | 365 (66.5%) | 391 (63.4%) | 332 (66.7%) | 1792 (66.4%) | 0.133^1^ |
| Nairobi | 22 (32.4%) | 131 (28.5%) | 177 (35.0%) | 184 (33.5%) | 226 (36.6%) | 166 (33.3%) | 906 (33.6%) |  |
| **Gender** |  |  |  |  |  |  |  |  |
| Female | 8 (11.8%) | 40 (8.7%) | 32 (6.3%) | 47 (8.6%) | 53 (8.6%) | 45 (9.0%) | 225 (8.3%) | 0.519^1^ |
| Male | 60 (88.2%) | 420 (91.3%) | 474 (93.7%) | 502 (91.4%) | 564 (91.4%) | 453 (91.0%) | 2473 (91.7%) |  |
| **Age** |  |  |  |  |  |  |  |  |
| Mean (SD) | 29.926 (5.253) | 31.530 (6.296) | 31.945 (6.356) | 32.158 (6.446) | 33.261 (6.954) | 33.845 (7.026) | 32.519 (6.667) | <0.001^2^ |
| Min - Max | 19- 39 | 20- 53 | 19- 54 | 19- 61 | 18- 66 | 18- 59 | 18- 66 |  |
| **Unstable housing** | |  |  |  |  |  |  |  |
| I am mobile | 15 (22.1%) | 90 (19.6%) | 112 (22.1%) | 112 (20.4%) | 116 (18.8%) | 29 (5.8%) | 474 (17.6%) | <0.001^1^ |
| I stay in one place | 53 (77.9%) | 370 (80.4%) | 394 (77.9%) | 437 (79.6%) | 451 (81.2%) | 469 (94.2%) | 2224 (82.4%) |  |
| **Ever imprisoned*** | |  |  |  |  |  |  |  |
| No | 0 | 0 | 37 (14.0%) | 79 (14.4%) | 100 (16.2%) | 93 (18.7%) | 309 (16.0%) |  |
| Yes | 0 | 0 | 228 (86.0%) | 470 (85.6%) | 517 (83.8%) | 405 (81.3%) | 1620 (84.0%) |  |
| Missing | 68 | 460 | 241 | 0 | 0 | 0 | 769 |  |
| **Years injecting** | |  |  |  |  |  |  |  |
| Median (Q1, Q3) | 3 (2, 6.250) | 2.000 (1.000, 5.000) | 3.000 (2.000, 6.000) | 3.000 (2.000, 6.000) | 4.000 (2.000, 7.000) | 4.000 (3.000, 7.000) | 4.000 (2.000, 6.000) | <0.001^2^ |
| Missing | 0 | 3 | 0 | 0 | 3 | 0 | 6 |  |
| **HIV-status** |  |  |  |  |  |  |  |  |
| Negative | 68 (100%) | 456 (99.1%) | 491 (97.2%) | 533 (97.1%) | 605 (98.1%) | 486 (97.6%) | 2639 (97.8%) |  |
| Positive | 0  (0.0%) | 4  (0.9%) | 14 (2.8%) | 16 (2.9%) | 12 (1.9%) | 12 (2.4%) | 58  (2.2%) |  |
| Indeterminate | 0 | 0 | 0 | 0 | 0 | 0 | 0 |  |
| Missing | 0 | 0 | 1 | 0 | 0 | 0 | 1 |  |
| **Times injected in last 30 days** | | |  |  |  |  |  |  |
| Mean (SD) | 64.941 (39.619) | 74.507 (34.852) | 71.962 (36.574) | 79.699 (31.893) | 75.596 (37.777) | 72.024 (38.958) | 74.636 (36.317) | 0.002^2^ |
| Median (Q1-Q3) | 60.000 (30.000, 90.000) | 60.000 (60.000, 90.000) | 90.000 (60.000, 90.000) | 90.000 (60.000, 90.000) | 90.000 (60.000, 90.000) | 90.000 (60.000, 90.000) | 90.000 (60.000, 90.000) |  |
| **Used previously used needle at last injection** | | | | | |  |  |  |
| No | 61 (89.7%) | 455 (98.9%) | 498 (98.4%) | 547 (99.6%) | 603 (97.7%) | 492 (98.8%) | 2656 (98.4%) |  |
| Yes | 7 (10.3%) | 5 (1.1%) | 8 (1.6%) | 2 (0.4%) | 14 (2.3%) | 6 (1.2%) | 42 (1.6%) |  |
| Don’t know | 0 (0.0%) | 0 (0.0%) | 0 (0.0%) | 0 (0.0%) | 0 (0.0%) | 0 (0.0%) | 0 (0.0%) |  |
| **OAT Accessed** | |  |  |  |  |  |  | < 0.001^1^ |
| **No** | 68 (100%) | 460 (100%) | 506 (100%) | 548 (99.8%) | 582 (94.3%) | 428 (85.9%) | 2592 (96.1%) |  |
| **Yes** | 0 (0.0%) | 0 (0.0%) | 0 (0.0%) | 1 (0.2%) | 35 (5.7%) | 70 (14.1%) | 106 (3.9%) |  |
| **NSP accessed** | |  |  |  |  |  |  | < 0.001^1^ |
| **No** | 68 (100%) | 441 (95.9%) | 135 (26.7%) | 64 (11.7%) | 97 (15.7%) | 57 (11.4%) | 862 (31.9%) |  |
| **Yes** | 0 (0.0%) | 19 (4.1%) | 371 (73.3%) | 485 (88.3%) | 520 (84.3%) | 441 (88.6%) | 1836 (68.1%) |  |

*Ever imprisoned was not asked in rounds 1 and 2; 1. Pearson’s Chi-squared test; 2. Kruskal-Wallis rank sum test.

**Supplementary Table 9**: Generalized Estimating Equations (GEE) logistic regression results for variables associated with outcome “used previously used syringe at last injection”. The odds ratio (OR) for opioid agonist therapy (OAT) access could not be estimated using this method due to separation (no one reported using a previously used syringe at last injection for individuals using OAT). Analysis conducted on complete cases (6,190 clusters with maximum size 6).

| **Variable** | **Unadjusted OR (95% CI)** | **Adjusted aOR (95% CI)** |
| --- | --- | --- |
| Accessed NSP in last 12 months(reference is no NSP) | 0.29 (0.23-0.37) | 0.30 (0.23-0.38) |
| Nairobi region (reference is Coastal region) | 2.44 (1.95-3.05) | 2.04 (1.60-2.60) |
| Male gender (reference is female) | 0.63 (0.47-0.83) | 0.70 (0.52-0.93) |
| Stably housed (reference is unstable housing) | 0.36 (0.29-0.44) | 0.55 (0.44-0.69) |
| Injecting duration in years | 1.00 (0.99-1.02) | 1.01 (0.99-1.02) |

NSP: needle and syringe programme

**Supplementary Table 10:** Hazard ratios (HR) for associations with HIV acquisition risk by Cox proportional hazards models.

| **Variable** | **Unadjusted HR (95% CI)** | **Adjusted aHR (95% CI)** |
| --- | --- | --- |
| Male gender (reference: female) | 0.29 (0.13-0.70) | 0.30 (0.12-0.74) |
| Nairobi region (reference: Coast) | 2.18 (1.09-4.34) | 2.16 (0.78-5.96) |
| NSP only in last 12 months (reference: neither) | 0.25 (0.09-0.70) | 0.23 (0.08-0.61) |
| OAT only in last 12 months (reference: neither) | < 10^-6^ | < 10^-6^ |
| Both NSP and OAT in last 12 months (reference: neither) | < 10^-6^ | < 10^-6^ |
| Stable housing (reference: unstable housing) | 0.48 (0.23-1.01) | 0.78 (0.25-2.38) |

OAT: opioid agonist therapy; NSP: needle and syringe programme

**Supplementary Figure 1**: Kaplan-Meier curves for becoming newly infected with HIV stratified by region (1A) and by gender (1B).

A

B
